# Simultaneous Inhibition of the HGF/MET and Erk1/2 Pathways Affect Uveal Melanoma Cell Growth and Migration

**DOI:** 10.1371/journal.pone.0083957

**Published:** 2014-02-13

**Authors:** Chandrani Chattopadhyay, Elizabeth A. Grimm, Scott E. Woodman

**Affiliations:** 1 Melanoma Medical Oncology, University of Texas, MD Anderson Cancer Center, Houston, Texas, United States of America; 2 Systems Biology, University of Texas, MD Anderson Cancer Center, Houston, Texas, United States of America; The Moffitt Cancer Center & Research Institute, United States of America

## Abstract

**Purpose:**

Nearly all primary uveal melanoma (UM) that metastasize involve the liver. Hepatocyte growth factor (HGF) is proposed to be an important microenvironmental element in attracting/supporting UM metastasis through activation of MET. The majority (>85%) of UM express mutations in the G-alpha proteins, that drive the MEK-ERK1/2 pathway. Thus, we proposed that the combination of MET and MEK inhibition would inhibit the growth and migration of G-alpha protein mutant versus non-mutant UM cells.

**Methods:**

Western-blots demonstrated the relative protein levels of ERK1/2 and MET in UM cells. Cells were treated with the small molecule inhibitors AZD6244 (MEKi) and/or MK-8033 (METi) and downstream markers evaluated. Further studies determined the effect of combination MEKi and METi treatment on cell growth, apoptosis and migration.

**Results:**

All G-alpha protein mutant UM cell lines express MET mRNA and protein. The level of mRNA expression correlates with protein expression. MEKi, but not METi treatment results in markedly reduced ERK1/2 phosphorylation. Either MEKi or METi treatment alone results in reduced cell proliferation, but only modest induction of apoptosis. The combination MEKi+METi results in significant reduction of proliferation in G-alpha protein mutant cells. UM cell migration was blocked by METi, but not MEKi treatment.

**Conclusions:**

MET protein expression showed no correlation with G-alpha protein mutation status. Combining MEKi with METi treatment has added benefit to either treatment alone in reducing G-alpha protein mutant UM cell growth. Combining METi with MEKi treatment adds the effect of limiting uveal melanoma cell migration.

## Introduction

In Western countries, uveal melanoma is diagnosed in approximately 4–11 cases per million people per year [1], [2]. Nearly 50% of patients with uveal melanoma develop metastases by 10 to 15 years after diagnosis, and the metastatic disease is universally fatal. The metastatic disease mortality rate remains unchanged despite advances in treating the primary eye tumor. More research into the biology of uveal melanoma is needed urgently to understand the critical clinically targetable pathways that will lead to improved patient outcomes.

The recent identification of activating mutations in the G-alpha protein from the *GNAQ* gene in uveal melanoma has provided a key insight into potential strategies in which to target uveal melanoma cell growth and survival [3], [4]. The mutations are somatic and occur in either amino acid sites R183 or Q209 turning *GNAQ* into a dominant oncogene with constitutive RAS/MEK/ERK1/2 signaling activation [5], [6]. However, we and others have shown that although small molecule MEK inhibitors can decrease cell growth in *GNAQ* mutant cells, MEK inhibition alone often fails to mediate significant apoptotic responses in these cells [4], [7]. In addition, similar somatic mutations in R183 or Q209 in the *GNA11* gene have been reported, and together with the *GNAQ*, represent approximately 85% of all primary uveal tumors.

It is known that uveal melanoma metastasizes to the liver via the hematogenous route, however the mechanism responsible for liver-predominant metastasis is unknown. Currently, there is no available systemic therapy for preventing or treating uveal melanoma metastases; so our focus on liver biology, and specifically on secreted growth factors like hepatocyte growth factor (HGF) is a high priority. The receptor for HGF, named c-MET (MET), has been found in uveal melanoma tumors and cells previously [8] and our own work on MET in N-Ras mutated cutaneous melanoma has prompted us to explore this system in more detail [9].

Prior studies have indicated that HGF may play an important role in mediating uveal melanoma growth and metastasis. Mallekarjuna et al., showed that primary uveal melanoma tumors that metastasize had higher levels of MET expression than tumors that did not metastasize [10]. In addition, a significant association between MET expression and uveal melanoma specific-mortality was noted. These investigators and others have also shown elevated MET levels in uveal melanoma cells within uveal melanoma tumors[8], [10]–[12].

In this study we investigate the effect of MEKi and/or METi treatment on *GNAQ* mutant and wild-type uveal melanoma cell lines. Our goal is to determine whether any biological basis exists for combined METi and MEKi treatments as a potential targeted therapy option, and whether the effects are more pronounced in mutant G-alpha protein cells or not.

## Materials and Methods

### Reagents

The small molecule MET inhibitor, MK-8033, was obtained under a material transfer agreement with Merck and Co (SRA # LS2009-00026397JW). AZD6244 was obtained from Selleckchem (Houston, TX, USA). Human HGF was purchased from R& D Systems (Minneapolis, MN). MK-8033 or AZD6244 were dissolved in dimethyl sulfoxide (DMSO) to prepare a stock solution of 10 mM, and then diluted as indicated in fresh medium. In all experiments, the final concentration of DMSO was <0.1%.

### Cells and Cell Culture

Melanoma cells were grown in RPMI 1640 medium supplemented with 10% fetal bovine serum.

All uveal melanoma cell lines used in this study were a generous gift of Martine Jager (Leiden University, Netherlands), whose laboratory generated the 92.1 cell line [13]. Mel202, Mel270, Mel 285, Mel 290, OMM2.3, and OMM2.5 originated from Bruce Ksander [14]. OCM1 and OCM3 originated from the laboratory of June Kan-Mitchell [15]. Cell line validation was accomplished by short random repeat (STR) DNA fingerprinting techniques and mutational analysis by MDACC Cancer Center Support Grant (CCSG)–supported Characterized Cell Line Core. Cell lines were validated by STR DNA fingerprinting using the AmpFSTR Identifier Kit (Applied Biosystems, Foster City, CA) according to manufacturer’s instructions. The STR profiles were compared to known ATCC fingerprints (ATCC.org), and to the Cell Line Integrated Molecular Authentication database (CLIMA) version 0.1.200808 (http://bioinformatics.istge.it/clima/). The STR profiles matched known DNA fingerprints or were unique [16].

### Western Blotting and Antibodies

Cells were lysed in a buffer containing 50 mM Tris (pH 7.9), 150 mM NaCl, 1% NP40, 1 mM EDTA, 10% glycerol, 1 mM sodium vanadate, and protease inhibitor cocktail (Roche Pharmaceuticals, Nutley, NJ). Proteins were separated by SDS-PAGE with 4–20% gradient gels (Bio-Rad Laboratories, Hercules, CA), transferred to a Hybond-ECL nitrocellulose membrane (GE Healthcare Biosciences, Piscataway, NJ), and blocked in 5% dry milk in PBS. The membrane was then incubated with primary and secondary antibodies, and target proteins were detected with ECL detection reagent (GE Healthcare Biosciences).

MET(C-12), PARP and beta actin antibodies were obtained from Santa Cruz Biotechnology, Phospho-Met antibody was purchased from Invitrogen, Phospho Erk1/2 and Erk1/2 antibodies were obtained from Cell Signaling Technology.

### Cell Proliferation Assays

Melanoma cells were plated at a density of 1×10^4^ cells/well in triplicate wells in a 24-well plate in RPMI 1640 growth medium, treated with MK-8033 (0 or 2 µM) and/or AZD6244 (0 or 25 nM) for 72 hours. MTT reagent [3-(4,5-dimethylthiazol-2-yl)-2,5-diphenyltetrazolium bromide] (Sigma-Aldrich, St. Louis, MO), dissolved in PBS, was added to a final concentration of 1 mg/mL. After 3 hrs, the precipitate formed was dissolved in DMSO, and the color intensity estimated in a MRX Revelation microplate absorbance reader (Dynex Technologies, Chantilly, VA) at 570 nm. The assays were done with two replicates for each condition.

### Cell Apoptosis Assay

Cells were treated with MK-8033 (0 or 2.5 µM) and/or AZD6244 (0 or 25 nM) for 72 hours, and prepared as a suspension of 1×106 cells/mL of PBS. After fixation with 90% ethanol for 1 hr, cells were centrifuged and stained with propidium iodide (PI) (Boehringer Mannheim, Indianapolis, IN) at a final concentration of 5 mg/mL PI and 10 mg/ml RNAse. DNA content and cell cycle phase were analyzed using a FACScan flow cytometer (Becton Dickinson, San Jose, CA).

### Cell Migration Assay

Cell migration assays were performed in Boyden chambers using uncoated filters (BD Biocoat control inserts, BD Biocoat, San Jose, CA). 2.5×10^5^ cells/well were plated in serum-free medium, with or without a four hour treatment of MK-8033, and the migration assay performed. Stained cells were photographed with a Nikon Eclipse TE2000-U microscope at 20X magnification using NIS Elements advanced research software. To quantify migration, the cells in each filter were counted from five independent fields under the microscope at 40X magnification and the mean cell number/field was calculated. Each assay condition was tested in 2 replicates.

### RNA Isolation

Total cellular RNA was extracted according to manufacturer’s instructions using a NucleoSpin RNA II kit (Macherey-Nagel, Bethlehem, PA).

### RNA Interference Assay

Cells were plated in 6 well plates (300,000/well), and after overnight incubation, the media was replaced by 900 uL of Opti-MEM (Life technologies#31985088). The cells were transfected with either non targeting siRNA (ThermoFisher#D-001210-03-20) or MET siRNA (Life Technologies#1299001), in quadruplet sets and equal molar amount. Un-transfected cells served as negative control. For each well 50 uL of Opti-MEM was mixed with 1 uL of Lipofectamine RNAiMAX (Life technologies# 13778-100) in a tube. In another tube 50 uL of Opti-MEM was mixed with 1 uL of each respective siRNA (20 uL stock). The two tubes were combined and incubated for 20 min at room temperature. Then this was layered over cells, to give a final concentration of 20 nM of each respective siRNA. After 4 hours, 1 mL of regular media was added to each well. The cells were incubated for 72 h and harvested for Western blot or used for cell proliferation assay (described in materials and methods section).

### Reverse Transcription PCR (RT-PCR)

To determine the mRNA levels of MET in uveal melanoma cells, we performed first-strand cDNA synthesis with 400 ng of total RNA using a GeneAmp RNA PCR kit (Applied Biosystems, Carlsbad, CA) according to the manufacturer’s protocol. A 2-µL cDNA product was used for each 25-µL PCR reaction t. The RT-PCR primers used to detect human MET and beta-actin were adapted from the publication by Fujita and Sugano [17]. The PCR protocol consisted of initial denaturation at 95°C for 5 min; 30 cycles of 95°C for 40 s, 55.5°C for 30 seconds, and 72°C for 60 s; primer extension at 72°C for 1 min; and a final extension at 72°C for 10 min. We analyzed 20 µL of PCR product on a 1% agarose gel.

## Results

### MET mRNA and Protein Expression in Uveal Melanoma Cell Lines

**Figure 1:**
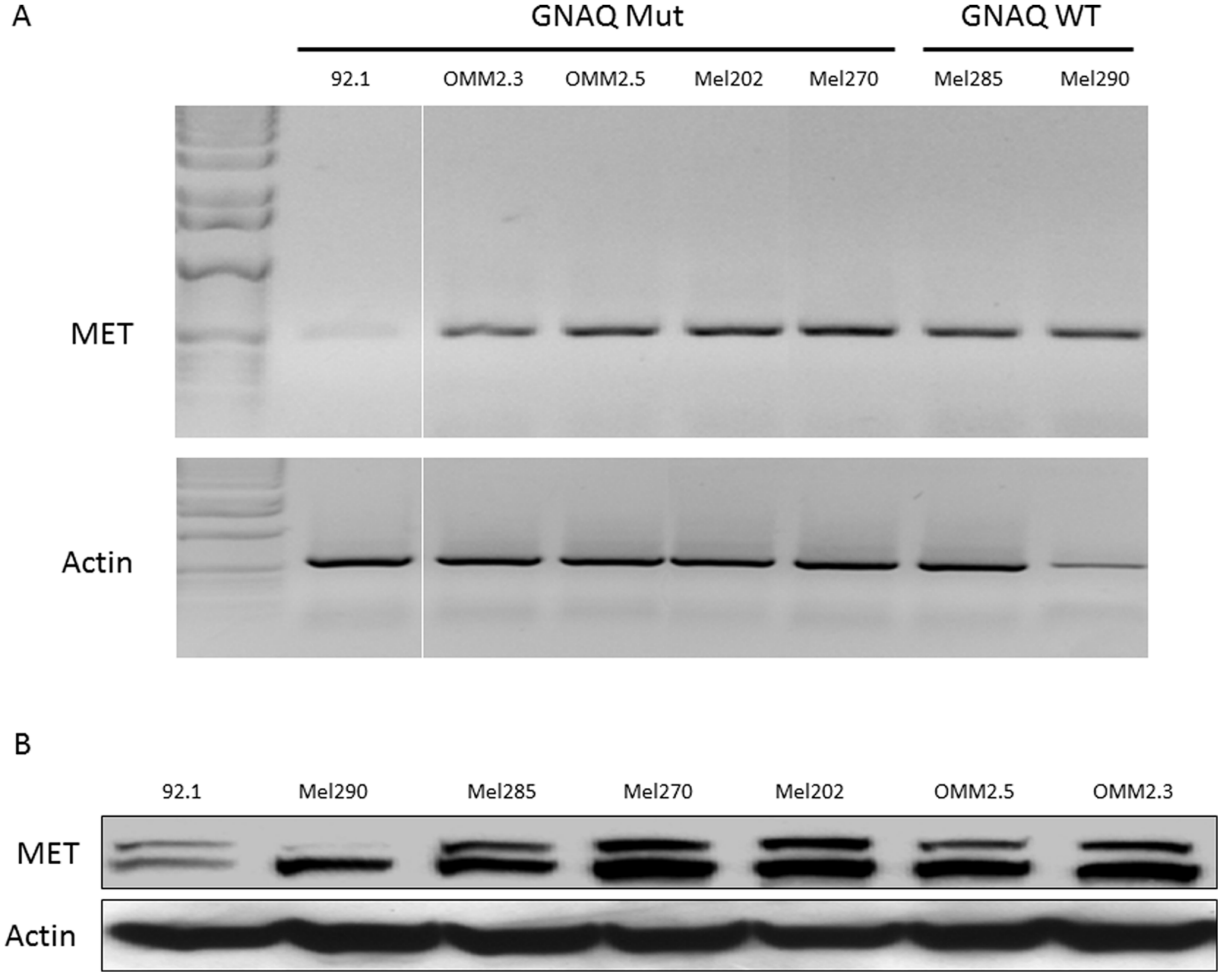
MET mRNA expression levels in *GNAQ* mutant and wild-type uveal melanoma cells. (A) MET mRNA expression (top panel) and actin mRNA expression (lower panel) as determined by RT-PCR. Images are from the same gel at the same exposure. (B) MET protein expression levels in *GNAQ* mutant and wild-type uveal melanoma cells. MET protein expression (top panel) and actin protein expression (lower panel) as determined by western-blot.

Total RNA was extracted from uveal melanoma cell lines and levels of MET mRNA transcript determined by RT-PCR (Figure 1A). All uveal melanoma cell lines, regardless of *GNAQ* mutation status, exhibited MET mRNA expression, although the 92.1 (*GNAQ* Q209L) cell line displayed far less MET mRNA transcript compared to the other cell lines. Each of these cell lines also demonstrated MET protein expression, irrespective of *GNAQ* mutation background (Figure 1B). Consistent with the lower MET transcript levels in the 92.1 cell line, the MET protein level was also lower in these cells. Of note, the MEL270, OMM2.3 and OMM2.5 cell lines are derived from the same individual [18], [19], and show similar MET mRNA and protein expression. Cell lines that have been stated to be of uveal melanoma origin, but for which we and others have documented the presence of the *BRAF* V600E mutation, [16] do not show significant MET protein expression despite displaying detectable MET transcript levels (Figure S1A&B).

### Effect of MEKi and/or METi Treatment on MET and Erk1/2 Phosphorylation in Uveal Melanoma Cells

**Figure 2:**
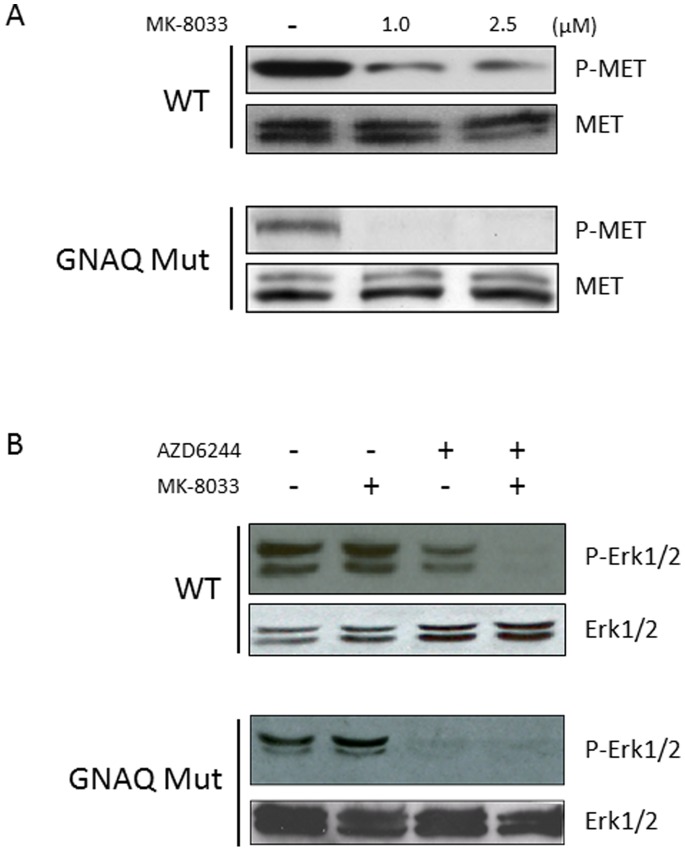
Effect of METi (MK-8033) treatment on MET phosphorylation in *GNAQ* mutant and wild-type uveal melanoma cells. (A) The upper panel demonstrates the effect of MK-8033 at 0, 1 and 2.5 µM on MET phosphorylation in WT uveal melanoma cells (MEL290), and the lower panel demonstrates the effect of MK-8033 on MET phosphorylation in *GNAQ* mutant cells (MEL202). Total MET protein expression is shown for each cell line under each condition. (B) Effect of METi (MK-8033) and/or MEKi (AZD6244) treatment on Erk1/2 phosphorylation in *GNAQ* mutant and wild-type uveal melanoma cells. The upper panel demonstrates the effect of MK-8033 and/or AZD6244 on Erk1/2 phosphorylation in WT uveal melanoma cells (MEL285), and the lower panel demonstrates the effect of MK-8033 and/or AZD6244 on Erk1/2 phosphorylation in *GNAQ* mutant uveal melanoma cells (MEL202).

**Figure 3:**
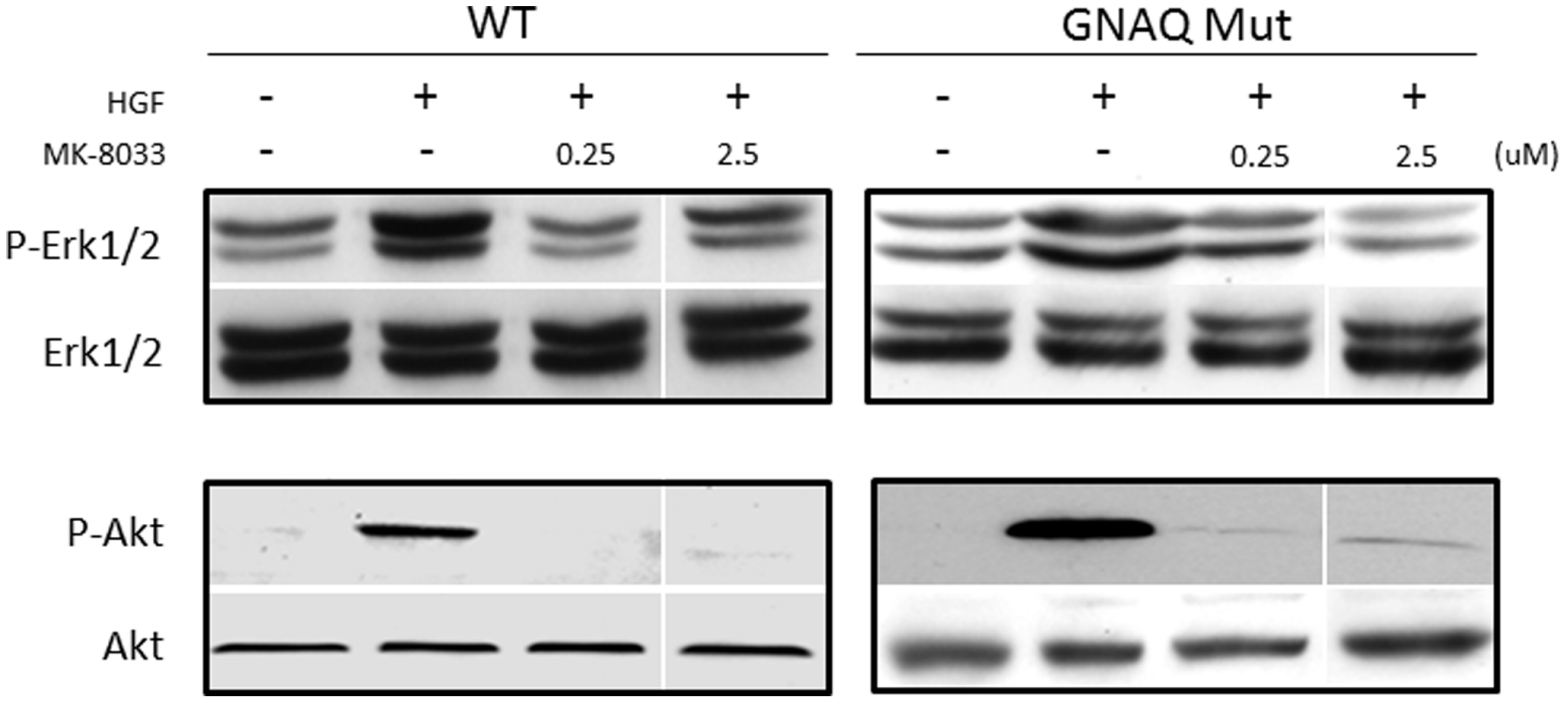
Effect of METi treatment on Erk1/2 and AKT phosphorylation in *GNAQ* mutant and wild-type uveal melanoma cells following stimulation with HGF (100/ng/ml). The effect of HGF on uveal melanoma cell Erk1/2 phosphorylation (upper panel) or AKT phosphorylation (lower panel) with or without METi treatment at 0.25 or 2.5 µM. Images are from the same blot at the same exposure.

We have previously demonstrated the capacity of the small molecule MEK inhibitor (AZD6244) to decrease ERK1/2 phosphorylation in uveal melanoma cell lines at low nanomolar concentrations [20]. To determine the capacity of the small molecule MET inhibitor (MK-8033) to inhibit MET phosphorylation in uveal melanoma cell lines with a *GNAQ* mutation versus no *GNAQ* mutation (hereafter “wild-type”), cells were treated with MK-8033 at either 0, 1 or 2.5 µM concentrations (Figure 2A). METi treatment reduced MET phosphorylation in both *GNAQ* mutant or wild-type cells by a relatively similar proportion, relative to baseline MET phosphorylation levels. To test the combined effect of MEKi and/or METi treatment, *GNAQ* mutant and wild-type cells were treated with AZD6244 (MEKi) at 25 nM and/or MK-8033 (METi) at 2.5 µM. MEKi treatment resulted in a marked decrease in Erk1/2 phosphorylation in both *GNAQ* mutant and wild-type cells, although complete inhibition was not observed in wild-type cells. METi treatment up to 2.5 µM failed to reduce Erk1/2 phosphorylation in either *GNAQ* mutant or wild-type cells. The combination MEKi+METi treatment recapitulated the effect of MEKi treatment alone in *GNAQ* mutant cells, but served to enhance the reduction of MEKi alone treatment in wild-type cells (Figure 2B). To further analyze the functional significance of MET expression in uveal melanoma, cells were treated with HGF (100 ng/ml), the ligand for MET. HGF treatment caused marked elevation of ERK1/2 phosphorylation which was decreased with co-treatment of low micro-molar concentrations of the METi, showing the efficacy of the METi at a HGF concentration higher than present in serum (Figure 3). Of note, METi treatment also extinguished the HGF induced phosphorylation of AKT.

### Effect of MEKi and/or METi Treatment on Uveal Melanoma Cell Proliferation

**Figure 4:**
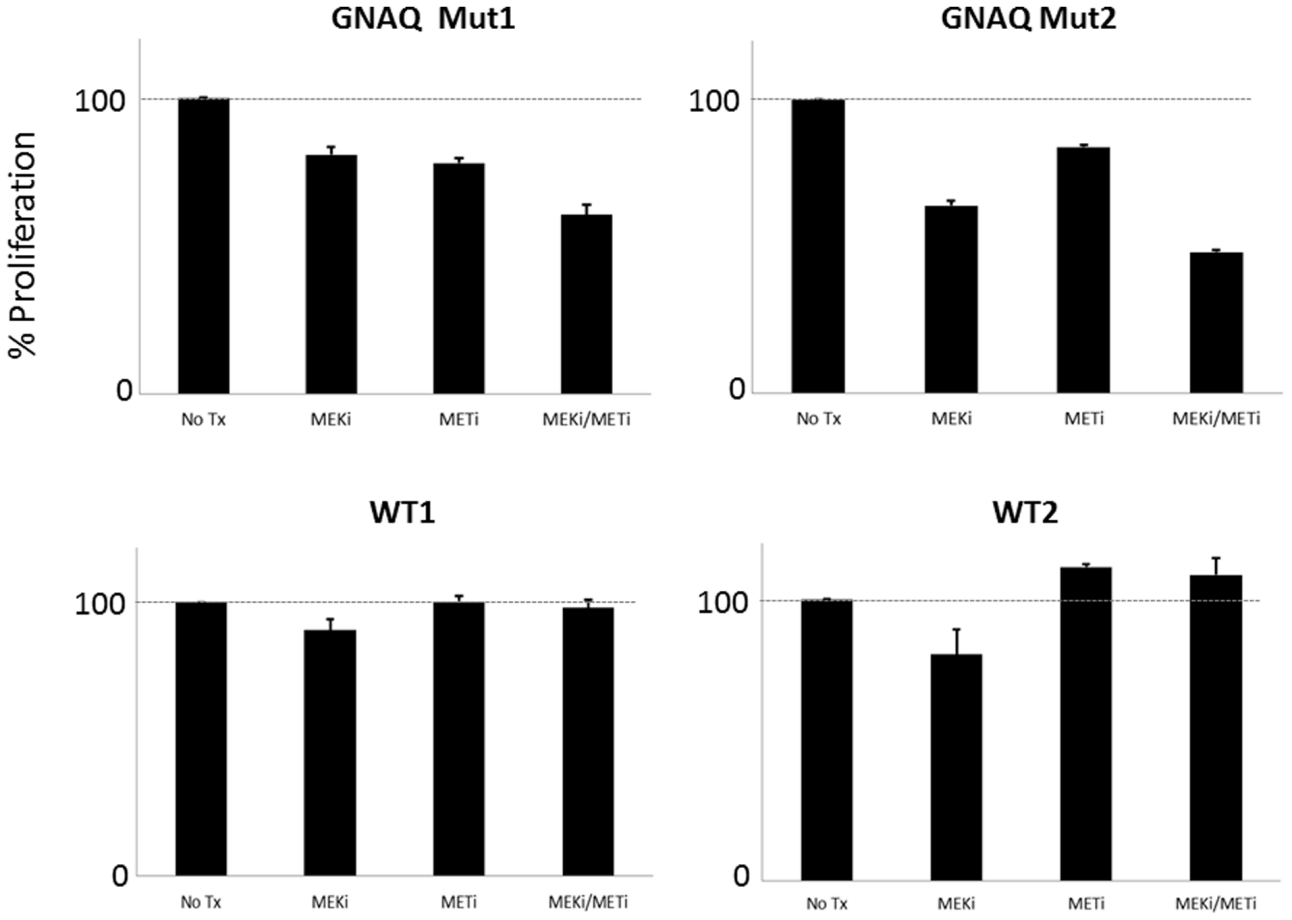
Effect of MEK and/or MET inhibition on the growth of *GNAQ* mutant versus wild-type uveal melanoma cells. The upper panel shows the effect of 25/or 2.5 µM METi treatment on two distinct *GNAQ* mutant cells, OMM2.3 (left) and MEL202 (right). The lower panel shows the results of the same treatment conditions in *GNAQ* wild-type cells, MEL285 (left) and MEL290 (right).

To determine the effect of MEK and/or MET inhibition on the growth of uveal melanoma, cells were treated with MEKi and/or METi (Figure 4). Relatively lower concentrations of each drug were used in order to optimize the potential of enhanced effects being observed with combination therapy. Treatment with either the METi or MEKi alone resulted in a noted decrease in cell proliferation in *GNAQ* mutant uveal melanoma cells. MEKi treatment resulted in a modest decrease in proliferation of wild-type cells, but no difference was observed after METi treatment in wild-type cells. The combination MEKi+METi treatment resulted in an enhanced inhibition of cell growth in *GNAQ* uveal melanoma cells, but not in wild-type cells.

Targeted siRNA knockdown of MET was performed to better determine the specific effect of MET reduction has on uveal melanoma cellular proliferation. The relative degree of MET knockdown, as determined by MET protein expression, was similar in mutant and wild-type cells (Figure S2A). The siRNA knockdown of MET resulted in a decrease in proliferation in all uveal melanoma cell lines, with a more pronounced effect being observed in mutant cells (Figure S2B).

### Effect of MEKi and/or METi Treatment on PARP Cleavage in Uveal Melanoma Cells

**Figure 5:**
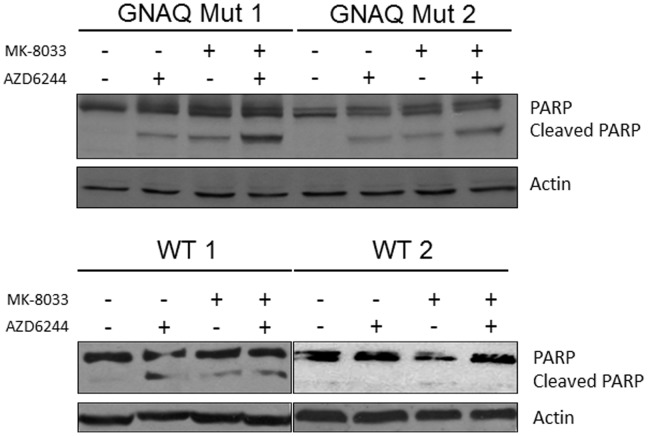
Effect of MEKi and/or METi treatment on PARP cleavage in uveal melanoma cells. The upper panel shows the effect of MEKi at 25/or METi at 2.5 µM on two distinct *GNAQ* mutant cells, OMM2.5 (left) and MEL202 (right). The lower panel shows the results in *GNAQ* wild-type cells, MEL285 (left) and MEL290 (right).

To determine the effect of MEK and/or MET inhibition on apoptosis in uveal melanoma, cells were treated with the MEKi and/or METi and PARP cleavage evaluated (Figure 5). Each cell line had little to no detectable cleaved PARP at baseline. Similarly increased levels of cleaved PARP were observed in *GNAQ* mutant uveal melanoma cells following either MEKi or METi treatment alone. The level of cleaved PARP was more enhanced by MEKi+METi combination treatment in *GNAQ* mutant cells. The MEL285 wild-type cell line (WT1) showed a modest increase in cleaved PARP to each agent alone, without a clear enhancement to combination treatment. The MEL290 wild-type cells (WT2) failed to show significantly increased levels of cleaved PARP with MEKi and/or METi treatment. Although low concentrations of each drug were employed in order to identify additive effects, the sub-G0 cell fraction in flow cytometry-based cell cycle analysis was observed to be slightly increased following combination AZD6244+ MK-8033 treatment in the *GNAQ* mutant cells, consistent with the cleaved PARP results (data not shown).

### Effect of MEKi and/or METi Treatment on Uveal Melanoma Cell Migration

**Figure 6:**
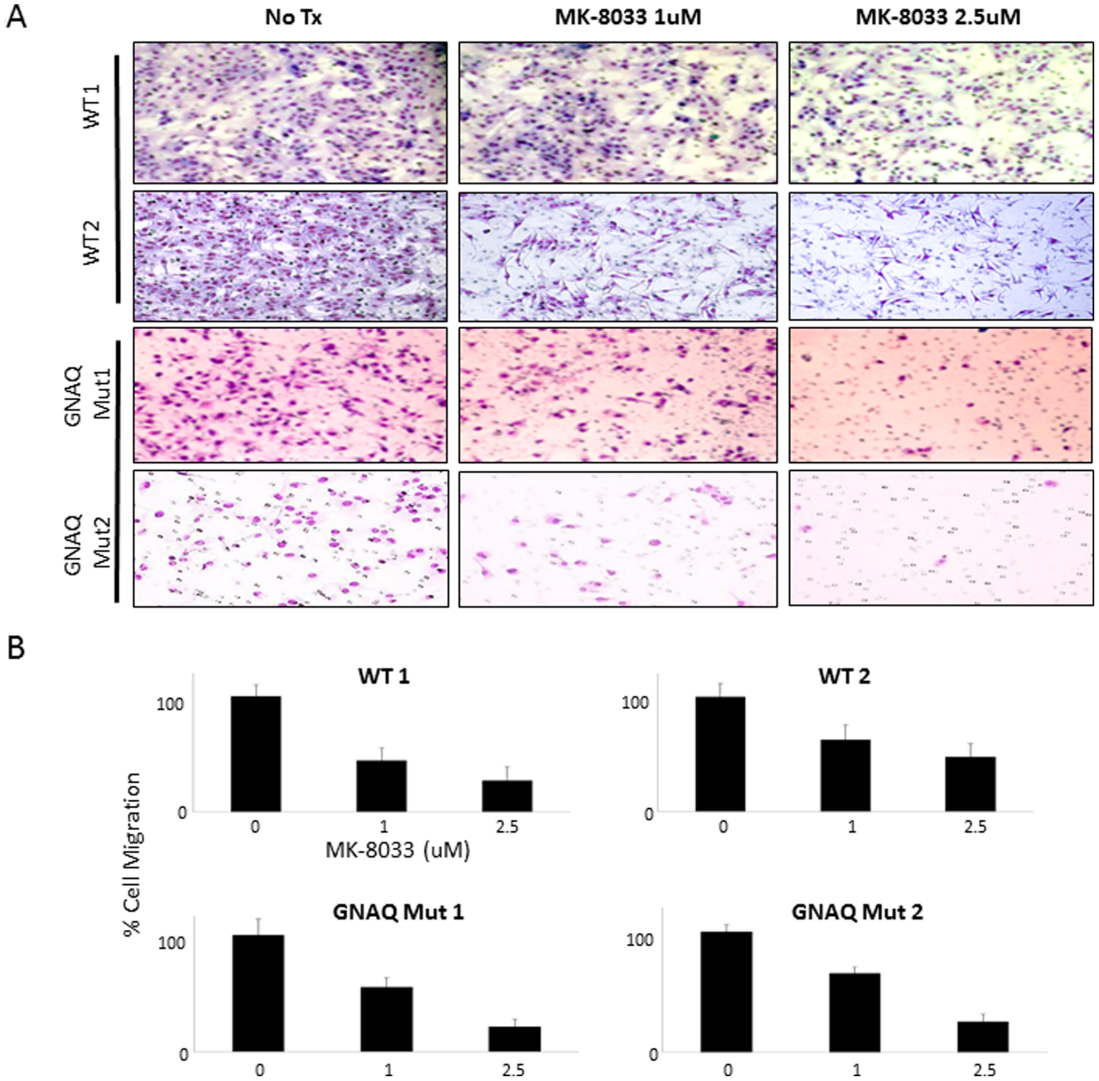
Effect of METi treatment on *GNAQ* mutant or wild-type uveal melanoma cell migration. (A) Representative images of distinct *GNAQ* wild-type (WT1 =  Mel285, WT2 =  Mel290) or mutant (Mut1 =  Mel270, Mut2 =  Mel 202) uveal melanoma cell line migration, stained following 0, 1 and 2.5 µM METi treatment. (B) Percent of *GNAQ* wild-type or mutant uveal melanoma cells that migrated following 0, 1 and 2.5 µM METi treatment.

To determine the effect of MEK and/or MET inhibition on cell migration, uveal melanoma cells with a *GNAQ* mutation were treated with MEKi and/or METi. Figure 6A shows images of stained migrated *GNAQ* mutant or wild-type cells following 0, 1 and 2.5 µM METi treatment. A marked reduction in cell migration was observed following METi treatment at micromolar concentration levels in all uveal melanoma cells, regardless of mutation or wild-type status (Figure 6B). Of note, METi treatment had no significant effect on cell migration in the *BRAF* mutant cell lines tested (Figure S3A), consistent with the lack of MET protein expression in these cells as shown in Figure S1B. MEKi treatment had no significant effect on cell migration in any of the cell lines tested (Figure S3B).

## Discussion

Approximately 85% of uveal melanoma cells harbor activating mutations in G-alpha proteins. When activated, these G-alpha proteins clearly signal through the MEK-ERK1/2 pathway. We have shown that small molecule inhibition of MEK is insufficient to drive cellular death in the majority of cells with *GNAQ/11* mutations, suggesting that other important pathways contributing to cell survival and are active in cells harboring these mutations [20].

In this study we demonstrate that uveal melanoma cells with a *GNAQ* mutation express the MET receptor at high levels. Employing small molecule inhibitors to both the MEK and the MET receptor, we show that MET inhibition enhanced the growth inhibitory effect of the MEKi. The METi had the added effect of inhibiting the migration of uveal melanoma cells regardless of *GNAQ* mutation or wild-type status in all cell lines expressing the MET receptor, suggesting that the migration inhibitory effects of METi are present across all uveal melanoma cell lines with MET expression. An interesting observation in the current study was that the METi alone treatment had growth inhibitory effects on *GNAQ* mutant cells, but not wild-type cells, at the concentration of METi used in this study. This observation is consistent with the findings of Abdel Rahman et al. who showed the MET inhibitor SU11274 to more markedly inhibit the proliferation of the uveal melanoma cell lines 92.1 and MEL202, compared to other cell lines tested [8]. It has since been determined that the 92.1 and MEL202 cell lines harbor *GNAQ* mutations, and the other cell lines treated in that study have neither *GNAQ* nor *GNA11* mutations [16].

At the doses used in the current study, METi treatment resulted in marked loss of MET phosphorylation in both *GNAQ* mutant and wild-type cells, although complete extinguishment of MET phosphorylation was not observed in wild-type cells. MET inhibition did not result in marked loss of ERK1/2 phosphorylation in either *GNAQ* or wild-type cells, thus the growth inhibiting effect of METi treatment in *GNAQ* mutant uveal melanoma cells may be mediated through a distinctly different signaling pathway. The MET receptor has been shown to signal through a myriad of other pathways (e.g., PI3K/AKT, STAT, Src, PLC) and is proposed to have cross-signaling effects on cell proliferation [21]. The elimination of the HGF induced PI3K phosphorylation following METi treatment (Figure 3) suggests a significant role for MET – PI3K/AKT signaling in growth and survival in uveal melanoma. Recent data supports a role for MET in uveal melanoma. The small molecule tyrosine kinase inhibitors XL814 or Crizotinib, which target MET, have been reported to show efficacy in mouse xenograft models of metastatic uveal melanoma [22], [23].

*GNAQ* mutant cells clearly demonstrated the induction of cleaved PARP with either of the MEKi or METi treatment doses employed. However, combination MEKi+METi treatment resulted in greater cleaved PARP levels than either treatment alone in these cells. Among the wild-type cells, the MEL 285 demonstrated low levels of cleaved PARP with all treatments, with no enhancement observed with combination treatment, and GNAQ wild-type MEL 290 failed to demonstrate evidence of cleaved PARP with MEKi and/or METi treatment. Thus, *GNAQ* mutant uveal melanoma cell lines appear more sensitive to the combination of MEK and MET. These findings are consistent with the studies of Wu et al., who failed to demonstrate cleaved PARP induction with two distinct MET inhibitors (PHA-665752 and PF-02341066) or with shRNA knockdown of MET in two cell lines that have neither *GNAQ* nor *GNA11* mutations (C918 and OCM1) [24]. Neither *GNAQ* nor *GNA11* mutant uveal melanoma cell lines were tested in that study. The observation in the current study that PARP cleavage occurred primarily in *GNAQ* mutant cells with MEKi and/or METi treatment suggests that the pathways driven by these molecules are important for cell survival, in a manner distinct from wild-type cells. However, only modest increases in the sub-G0 fraction of cells was noted, indicating the presence of counteracting anti-apoptotic mechanisms [25].

Two prior studies that investigated the use of single agent MET inhibitors, and used cell lines that did not harbor *GNAQ* mutations (e.g., OCM1, OCM3, OCM8, C918), but rather have *BRAF* mutations (published and unpublished data) [8], [24]. Using standard sequencing techniques *BRAF* mutations are not observed in primary uveal melanoma tumors, although two groups have identified *BRAF* mutations in a small number of primary uveal melanoma samples using more sensitive techniques [26], [27]. The clinical relevance of these findings awaits further investigation.

Prior to this study it was not known whether combined inhibition of MEK and MET caused further growth inhibition in G-alpha protein mutant uveal melanoma cells or whether the combination treatment further diminished the migration in these cells. Our data show that combined treatment of uveal melanoma cells with small molecule MEK and MET inhibitors simultaneously enhances the growth inhibitory effect of each agent in a *GNAQ* mutation-dependent manner, and yet the small molecule METi also served as a powerful blocker of migration in both *GNAQ* mutant and wild-type cells. This study greatly enhances our knowledge of the role played by the MET pathway in uveal melanoma cells, with new information proposing the combination treatment of small molecule MEK and MET inhibitors in cells harboring G-alpha protein gene mutations. Thus a combination of MEK and MET inhibitors can control uveal melanoma cell growth and migration better than individual treatment with each of these inhibitors alone.

Finally, the data presented in this paper may also be placed in the greater context of primary drug resistance given the observation by Straussman et al, that HGF derived from stromal cells results in reactivation of the mitogen-activated protein kinase (a.k.a., Erk1/2) and phosphatidylinositol-3-OH kinase (PI(3)K)-AKT signaling pathways, in *BRAF* inhibitor treated cutaneous *BRAF* mutant melanoma cells [28]. Likewise, the HGF-MET axis may play an important role in mediating resistance to MEK inhibition, and thus combination MEKi and METi treatment would be a rationale strategy under these conditions.

## Supporting Information

Figure S1 **MET mRNA expression levels in** ***BRAF*** **mutant uveal melanoma cells.** (A) The top panel shows MET mRNA expression and the lower panel shows actin mRNA expression as determined by RT-PCR. Images are from the same gel at the same exposure. (B) MET protein expression levels in *BRAF* mutant uveal melanoma cells. The top panel shows MET protein expression and the lower panel shows actin protein expression as determined by western-blot.
(TIF)

Figure S2 **Effect of siRNA knockdown of MET protein and cell viability.** (A) Effect of non-targeting versus MET targeting siRNA on MET protein level as determined by western-blot. (B) Effect of MET targeting siRNA on cell viability relative to non-targeting siRNA control.
(TIF)

Figure S3 **Effect of METi treatment on** ***BRAF*** **mutant uveal melanoma cell migration.** (A) Representative images of migrated *BRAF* mutant (OCM1 or OCM8) uveal melanoma cells stained following 0 or 1 µM METi treatment. (B) Effect of MEKi treatment on *GNAQ* wild-type or mutant uveal melanoma cell migration. Images of migrated wild-type (WT1 =  Mel285, WT2 =  Mel290) or mutant (Mut1 =  Mel270, Mut2 =  Mel 202) GNAQ uveal melanoma cells stained following 0 or 50 nM MEKi treatment.
(TIF)
