# Supplementary figures and images for: Simultaneous Inhibition of the HGF/MET and Erk1/2 Pathways Affect Uveal Melanoma Cell Growth and Migration
Source: PLoS One. 2014 Feb 13;9(2):e83957. doi: 10.1371/journal.pone.0083957 (PMC3923717; doi:10.1371/journal.pone.0083957)

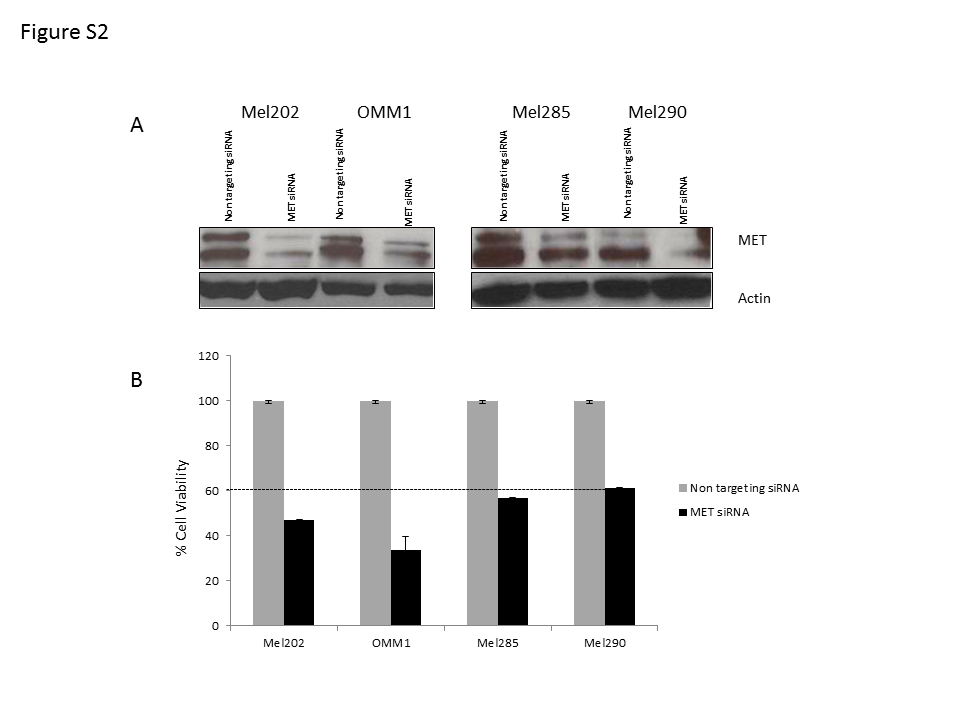

Supplement: Figure S2 — Effect of siRNA knockdown of MET protein and cell viability. (A) Effect of non-targeting versus MET targeting siRNA on MET protein level as determined by western-blot. (B) Effect of MET targeting siRNA on cell viability relative to non-targeting siRNA control. (TIF) [file pone.0083957.s002.tif]

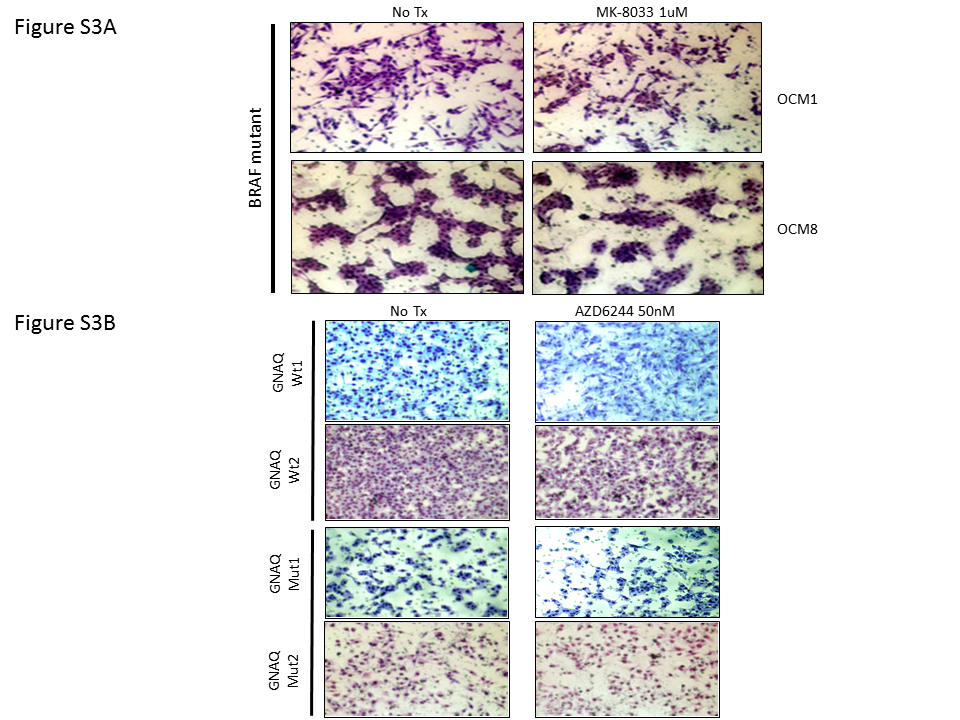

Supplement: Figure S3 — Effect of METi treatment on BRAF mutant uveal melanoma cell migration. (A) Representative images of migrated BRAF mutant (OCM1 or OCM8) uveal melanoma cells stained following 0 or 1 µM METi treatment. (B) Effect of MEKi treatment on GNAQ wild-type or mutant uveal melanoma cell migration. Images of migrated wild-type (WT1 = Mel285, WT2 = Mel290) or mutant (Mut1 = Mel270, Mut2 = Mel 202) GNAQ uveal melanoma cells stained following 0 or 50 nM MEKi treatment. (TIF) [file pone.0083957.s003.tif]
